# The role of social norms, intergroup contact, and ingroup favoritism in weight stigma

**DOI:** 10.1371/journal.pone.0305080

**Published:** 2024-06-20

**Authors:** Erin C. Standen, Andrew Ward, Traci Mann

**Affiliations:** 1 Department of Psychology, University of Minnesota, Minneapolis, Minnesota, United States of America; 2 Department of Psychology, Swarthmore College, Swarthmore, Pennsylvania, United States of America; Polytechnic Institute of Coimbra: Instituto Politecnico de Coimbra, PORTUGAL

## Abstract

Although average body size in the U.S. has increased in recent decades, stigma directed at individuals with higher weight has not diminished. In this study, we explored this phenomenon by investigating the relationship between people’s perceived social norms regarding higher weight and their reported levels of weight bias (i.e., anti-fat attitudes). Our predictions for perceived social norms drew on the concepts of intergroup contact and ingroup favoritism, which were also probed in this study. We hypothesized that both greater descriptive norms and more favorable injunctive norms regarding higher weight would be associated with lower reported weight bias. Individuals’ quantity and quality of social contact with people with higher weight were also predicted to be associated with lower weight bias. Finally, we predicted that individuals who perceived themselves as heavier would display ingroup favoritism (i.e., report less weight bias). Participants (*N* = 272) from the United States completed a set of online questionnaires about their perceived social norms, social contact with people with higher weight, and explicit weight bias. We found support for each of these pre-registered predictions (*p*s < 0.03), and *post hoc* analyses revealed that quality, but not quantity, of social contact with individuals with higher weight was an important predictor of lower weight bias. Together, these findings provide insight into the social psychology of weight bias and help to lay a theoretical foundation for future efforts to reduce weight stigma.

## Introduction

Rates of having a high body weight have increased over the last several decades in the United States [1, 2], and during this time, people with higher weight have become more widely represented in the news media, retail campaigns, and advocacy sectors. Plus-size models appear more regularly in advertisements, major fashion retailers offer a wider range of sizes, and well-known athletes, actors, and musicians (e.g., Serena Williams, Jameela Jamil, Lizzo, James Corden) have publicly denounced anti-fatness [3, 4]. Likewise, organizations and movements dedicated to fat acceptance and protecting the rights of people in larger bodies (e.g., National Association to Advance Fat Acceptance) continue to grow their membership. Despite these positive developments, weight stigma remains prevalent [5, 6], and may even have increased over the past two decades [7–9].

In using the term “weight stigma,” we refer to the negative weight-related attitudes, beliefs, and stereotypes that people hold regarding people with higher body weight [10]. Weight stigma might have been expected to have decreased in recent years, given that, for several other traits or identities, stigma has indeed diminished as the relevant characteristic has become more visible and widespread. For instance, as members of the LGBTQ+ community have become more prominent and visible in the United States, rates of explicit antigay bias have decreased substantially [11], leading to a level of acceptance (e.g., legalized gay marriage) that may not have seemed possible a few decades ago. Indeed, some formal interventions intended to reduce prejudice capitalize on this phenomenon by emphasizing the commonness of a stigmatized trait, identity, or experience. For example, in an effort to normalize treatment-seeking behaviors, several campaigns aimed at reducing the stigma surrounding mental illness have emphasized its prevalence [12, 13].

In the present research, we explored why increased visibility of individuals with higher body weight and increased normativity of larger bodies have not led to substantial reductions in weight stigma. Based on standard Body Mass Index (BMI) cut-offs, only a statistical minority of Americans are in the so-called “normal” weight category. In fact, being categorized as “overweight” or “obese” is actually the norm, as these are the most common BMI categories: approximately 42% of Americans are categorized as obese (i.e., BMI ≥ 30 kg/m^2^) and 30% are categorized as “overweight” (i.e., BMI between 25 kg/m^2^ and 30 kg/m^2^; [1, 14]). In the context of this work, we focused on norms surrounding obesity, rather than norms surrounding overweight, as the former categorization is more stigmatized [15, 16]. Moreover, it is important to note that although BMI is commonly used by medical professionals and epidemiologists to classify individuals by body size, there are many ethical and methodological concerns with its origin and continued use, particularly when employed as an indicator of health [17–19]. In this paper, we use the term “obesity” to refer to the concept of having a body weight in the corresponding BMI category, but we note that many scholars and activists advocate for the use of other, less pathologizing terms (e.g., higher weight, fatness; [20–22]). Whenever not directly pursuant to the wording of questions posed to participants in this study, we use the term “higher weight” or “having a high body weight” in place of “obesity.”

In the context of the study presented here, it is instructive to consider the distinction between descriptive and injunctive norms [23]. A descriptive norm refers to how *common* a characteristic or behavior is, whereas an injunctive norm refers to how socially *accepted* a characteristic or behavior is. Research on these forms of norms has been conducted in a variety of contexts (e.g., preventing littering, conserving electricity, reducing alcohol consumption) and has generally found that individuals will alter their behavior and beliefs to align with a perceived descriptive norm that implies a behavior is commonly engaged in or a perceived injunctive norm that implies a behavior is generally accepted [23–27]. Typically, social norms have been applied to behaviors, but some work has used them to understand perceptions of characteristics [28–30]. In this study, we focused on social norms of having a higher weight. Although having a higher weight has become more objectively common in recent years [1, 2], as well as perceived to be more common [30, 31], perceptions of societal disapproval remain high [5, 16, 29] and may have even increased over time [7, 9]. To explore this disconnect, we turned to the social psychological literature on intergroup contact and ingroup favoritism.

### Social norms, contact, and ingroup favoritism

Although there is relatively little research that explores the relationship between each type of social norm and weight bias, predictions can be made based on two well-established social psychological findings: the effects of contact on intergroup bias and the phenomenon of ingroup favoritism [32, 33]. Here, we describe the existing research on contact theory and ingroup favoritism in the context of weight bias. We then expand on these findings in order inform our predictions regarding the relationship between social norms and weight bias.

#### Contact theory and weight bias

Contact theory holds that stereotypes, prejudice, and discrimination exhibited by individuals in one group toward another group can be reduced through direct face-to-face contact between members of the groups [33]. Numerous studies have demonstrated support for this claim [34, 35]. Although direct contact alone is not always sufficient to reduce all forms of bias, it is considered a key feature and has been used successfully in numerous interventions involving interacting groups, particularly those based on race or ethnicity. Indeed, some studies have found that simply imagining contact can improve relations between certain groups [36]. Allport [33] described several conditions for optimal contact (e.g., equal status, common goals). Although meta-analytic evidence suggests that, while useful, these conditions are not necessary for contact to be effective [37], we selected a measure of contact quality that addresses these conditions [38], see *Measures*.

Research on the efficacy of contact for reducing weight stigma, however, is somewhat limited. Two survey studies found that positive contact with individuals with higher weight was related to lower weight bias [39, 40]. One of these studies also found that negative contact was related to greater weight bias [39]. Another study found mixed evidence for the bias-reducing benefits of contact, such that contact with heavier friends was related to lower weight bias, whereas greater exposure to higher-weight people in daily life actually predicted increased weight bias [41]. It is possible, of course, that the “contact” in this latter instance may not have been as high quality, in terms of Allport’s optimal conditions, as contact with a friend, but those characteristics were not measured in the study. Taken together, this evidence nevertheless provides some support for the contact hypothesis in the context of weight. In addition, some pertinent evidence can be found in a laboratory study in which, in addition to a “no contact” control condition, participants were randomly assigned to engage in direct contact with a person with higher weight, imagine such contact, or watch another person engage in contact [42]. Participants who engaged in direct contact with a person with higher weight reported less weight bias than did participants who had no contact with a person with higher weight (Cohen’s *d* = 0.92), who imagined such contact (*d* = 0.71), or who watched another person engage in such contact (*d* = 0.93), supporting the hypothesis that direct contact is indeed beneficial in terms of reducing weight bias.

#### Predictions regarding descriptive norms and weight bias

Based on the general predictions of contact theory, the more contact individuals have with people with higher weight, the less weight bias they should hold. Of course, the more people have a higher weight, the greater the likelihood that any individual will interact with such people, and the greater the likelihood that individuals will bring such instances to mind when considering how common it is to have a high body weight [43]. Indeed, a recent study found a significant negative relationship between national prevalence of obesity in participants’ country of residence and weight bias [41]. In the present study, we hypothesized a similar pattern for perceptions of obesity prevalence (i.e., descriptive norms); specifically, we predicted that participants who perceived that having a higher weight as more common would have lower levels of explicit weight bias.

#### Ingroup favoritism and weight bias

Drawn from social identity theory, the concept of ingroup favoritism holds that people are motivated to evaluate social groups with which they identify (i.e., their ingroup) more favorably than other groups [34, 44]. This phenomenon has been documented hundreds of times in social psychology studies [45]. Individuals generally prefer their ingroups to other groups, evaluate their ingroups more positively, and allocate more rewards to their own group [32]. These groups do not need to be long-lasting, meaningful, or cohesive. Indeed, many studies have been conducted using so-called minimal groups―groups that had just been formed through random processes and could not possibly be perceived by study participants as meaningful or cohesive [46]. And yet, individuals still prefer these groups to others.

Despite the substantial evidence for ingroup favoritism across many types of groups, the literature on ingroup favoritism among people with higher weight is mixed. In terms of research study findings, a negative correlation between BMI and weight bias would suggest ingroup favoritism, with people with higher BMIs reporting less weight bias. However, several studies have found no correlation between those variables [47–50]. Four other studies did find a negative correlation, suggesting an ingroup bias among individuals with higher weight [39, 51–53]. In one of those studies [51], absolute attitude scores were not reported, but in two others [52, 53], the average attitude level among higher-weight participants still indicated a preference for thin individuals over heavier ones. Therefore, only one study [39] clearly showed ingroup favoritism in terms of both the predicted negative correlation and absolute levels of explicit weight bias below the midpoint on the relevant attitude scale.

One possible explanation for the aforementioned mixed pattern of results involving explicit measures is that individuals with high BMIs may not necessarily perceive themselves as heavy. Likewise, some people with BMIs in the “normal” range may perceive themselves as heavy. Many studies have found evidence for discrepancies between people’s perceived weight and their objective weight status [54–56]. These sorts of discrepancies are critical to consider because a central concept of social identity theory is that individuals are motivated to favorably evaluate their *perceived* ingroups, rather than categorizations ascribed by other people. People’s perceived weight could be expected to reveal a stronger relationship with their level of weight bias than their actual BMI does, but the one study that examined this link found that perceived weight had a weaker relationship to weight bias than did actual BMI [51]. Nevertheless, when exploring these relationships, the extant literature and core concepts of social identity theory suggest that it is worthwhile to consider an individual’s perceived weight status.

#### Predictions regarding injunctive norms and weight bias

In terms of weight, the phenomenon of ingroup favoritism suggests that individuals who perceive themselves as heavy should express lower weight bias compared to people who do not perceive themselves as heavy. This idea aligns with injunctive norms regarding higher weight, as an individual may be hesitant to favor their own group if they find the defining feature of the group to be socially unacceptable (see [47]). Therefore, one might expect that positive injunctive norms regarding obesity (i.e., greater beliefs that having a high weight is socially accepted) would be inversely related to weight bias, particularly among individuals who identify as heavy.

### The present study

The overarching goal of this work was to document how social norms pertaining to obesity (i.e., descriptive norms and injunctive norms) relate to weight bias. Because we situated our predictions within the context of other social psychological concepts known to relate to weight bias, we also probed for effects of contact and ingroup favoritism within our sample.

In the present study, we measured participants’ perceived descriptive norms with regard to obesity (i.e., how common people believed it was to have a high weight), and we examined the relationship between this belief and their levels of weight bias. We expected that participants who reported descriptive norms that indicated they perceived a greater prevalence of obesity in the population would report less weight bias (Hypothesis 1). Next, we measured both the quantity (Hypothesis 2) and quality (Hypothesis 3) of contact that participants indicated they experienced with people with higher weight. For both, we expected higher levels to predict lower weight bias. Because people may have more opportunities to interact with people with higher weight when obesity is more common, we also hypothesized that the perceived descriptive norms reported by participants regarding obesity would be significantly positively related to their quantity of contact with people with higher weight (Hypothesis 4).

We also assessed injunctive norms concerning obesity (i.e., how socially accepted participants believe it is to have a high weight), and we hypothesized that those indicating more favorable injunctive norms would report less weight bias (Hypothesis 5). In order to gauge ingroup favoritism, we asked participants to report their perceived weight; we hypothesized that those individuals who perceived themselves as heavier would display lower levels of weight bias (Hypothesis 6). To further explore this phenomenon, we measured participants’ ingroup identification, that is, the degree to which they identified with their perceived weight category (i.e., thin, average, or heavy). Based on prior work on ingroup identification and intergroup bias [57, 58], we hypothesized that among individuals who perceived themselves as heavy, ingroup identification would be negatively related to weight bias (Hypothesis 7), suggesting that those who identify more strongly with their ingroup show greater ingroup favoritism. Finally, we investigated whether higher-weight individuals who believed that having a high body weight is socially acceptable would be more likely to report identification with the relevant ingroup. In other words, we examined whether, among individuals who perceived themselves as heavy, perceived injunctive norms would be positively related to their reports of ingroup identification (Hypothesis 8).

It important to note that, although our research design precluded the drawing of causal conclusions, this investigation was intended to further the field’s understanding of why weight stigma is not diminishing even as having a higher weight becomes more common. Moreover, by investigating how the social-psychological principles of contact and ingroup favoritism relate to descriptive and injunctive norms regarding obesity, we aimed to provide new insight into how these concepts may operate uniquely in the context of weight stigma.

## Methods

A total of 434 participants from the United States were recruited via Prolific, a commonly used source of paid research participants. We over-sampled to account for any participants who failed our attention check (see *Measures*), which left 272 participants for analysis. This target sample size was based on a power analysis conducted using G*Power (version 3.1). We anticipated a small effect size (*f*^2^ = 0.03) and sought to achieve a power of 0.8 with an alpha of 0.05, requiring a minimum of 264 participants. Most participants were white (74.91%) and female (69.37%), and ages ranged from 18 to 69 (*M* ***=*** 29.14, *SD* = 10.97). For full demographics, see Table 1.

**Table 1:** Demographics of research sample.

Demographic	N (%)
Sex	
Male	83 (30.63%)
Female	188 (69.37%)
Race/ethnicity	
White	203 (74.91%)
Black or African American	16 (5.90%)
Asian or Pacific Islander	15 (5.53%)
Hispanic or Latinx	16 (5.90%)
Multiple races or ethnicities	21 (7.75%)
Region of the United States	
Southeast	72 (26.57%)
Northeast	64 (23.62%)
West	57 (21.03%)
Midwest	47 (17.34%)
Southwest	31 (11.44%)
Body Mass Index (kg/m^2^)	
Underweight (< 18.5)	14 (5.51%)
Normal weight (18.5–24.99)	131 (51.57%)
Overweight (25–29.99)	57 (22.44%)
Obese (≥ 30)	52 (20.47%)

*Note*. Some participants declined to provide demographic information.

After providing written informed consent, participants completed measures assessing their perceived descriptive and injunctive norms regarding obesity, as well as their level of contact with people with higher weight, their ingroup identification, and their degree of explicit weight bias. They also reported their perceived weight and supplied demographic information. Finally, because some research has linked weight bias to political conservatism [47], participants were asked to indicate their political orientation. Compensation for participants was based on a rate of $6.50/hour, in accordance with Prolific’s fair wage policy. All study procedures were approved by the Institutional Review Board at the University of Minnesota (#STUDY00007777), and data collection took place between July 13, 2021 and July 29, 2021.

### Measures

#### Descriptive norms regarding obesity

The measure of perceived descriptive norms was adapted from the measure used by Primack and colleagues [59]. Participants were asked to estimate what percentage (in 10-percent increments) of people are classified as obese: (1) in the United States, (2) in their region of the United States, and (3) in their community. These estimates were averaged to create an index of descriptive norms regarding obesity (*α* = 0.88).

#### Injunctive norms regarding obesity

The measure of perceived injunctive norms with regard to obesity was also adapted from the measure used by Primack and colleagues [59]. Participants rated the degree to which they agreed with four statements, including the following: “Most people in the United States disapprove of being obese,” and “Most people in my community disapprove of being obese.” Response options ranged from 1 (*strongly disagree*) to 7 (*strongly agree*). Responses were reverse coded such that higher scores would indicate greater beliefs that having a high body weight is socially accepted. Scores were averaged to create a single measure of injunctive norms (*α* = 0.60).

#### Contact quantity

Contact quantity was assessed using a modified version of the General Intergroup Contact Quantity and Quality Scale–Contact Quantity Subscale [38]. Participants reported how much contact they had with people who are obese (1) at work or school, (2) as neighbors, and (3) as close friends, using a scale from 1 (*none at all*) to 7 *(a great deal*). They also reported how often they have (4) engaged in informal conversations with people who are obese and (5) visited the homes of people who are obese, using a scale from 1 (*not at all*) to 7 (*very often*). Scores on these five items were averaged to create an index of contact quantity (*α* = 0.89).

#### Contact quality

Contact quality was assessed using a modified version of the General Intergroup Contact Quantity and Quality Scale–Contact Quality Subscale [38]. Participants reported the extent to which they have experienced contact with people who are obese as (1) equal, (2) voluntary, (3) superficial, (4), pleasant, and (5) cooperative on scales ranging from 1 to 7. Scores for “superficial” were reverse coded and then scores were averaged so that higher scores indicated higher quality of contact (*α* = 0.79).

#### Perceived weight

Perceived weight was assessed by asking participants to rate themselves on the following scale: (1) very thin, (2) moderately thin, (3) slightly thin, (4) average, (5) slightly heavy, (6) moderately heavy, (7) very heavy. This method of measuring self-perceived weight is considered a standard approach in the study of weight stigma [60–63].

#### Ingroup identification

Participants’ levels of ingroup identification with their perceived weight category (collapsed into “thin,” “average weight,” and “heavy”) was assessed using four items adapted from those used by Crisp and Beck [64]. Participants answered the following questions on a scale from 1 (*not at all*) to 9 (*very much*), with their own perceived weight responses from earlier in the survey used in the question stems: (1) “I identify with people who perceive themselves as [participants’ perceived weight category],” (2) “Being [participants’ perceived weight category] is an important part of who I am,” (3) “I feel strong ties with people who perceive themselves as [participants’ perceived weight category],” and (4) “I feel a strong sense of solidarity with people who perceive themselves as [participants’ perceived weight category].” Responses to these four items were averaged to create an index of ingroup identification with one’s perceived weight category (*α* = 0.77).

#### Explicit weight bias

Explicit weight bias was assessed using the dislike subscale of the Anti-Fat Attitudes Questionnaire [47], a well-validated and widely used measure of weight bias [39, 40, 65]. Example items include: “I really don’t like fat people much,” and “Fat people make me somewhat uncomfortable.” Response options ranged from 0 (*Very Strongly Disagree*) to 9 (*Very Strongly Agree*), and higher scores indicate greater dislike. Responses were averaged to form a single anti-fat attitudes score (*α* = 0.91). One of the items from this scale, “I don’t have many friends who are fat,” overlaps conceptually with our measures of contact. Therefore, in analyses that use measures of contact to predict explicit weight bias, this item was dropped. The modified version also exhibited excellent reliability (*α* = 0.94).

#### Political orientation

Participants were asked to rate their political beliefs on a 7-point scale from (1) *Extremely Liberal* to (7) *Extremely Conservative*, a single-item measure often used to assess political orientation [66, 67].

#### Demographic information

Participants reported their age, sex, race/ethnicity, height (in inches), and weight (in pounds). BMI was calculated using the standard formula (weight in kilograms divided by height in meters squared), after conversion of units. Participants were also asked to indicate the region of the country they live in, from the following options: Northeast, Southeast, Midwest, Southwest, and West.

#### Attention check

Because data quality from online survey platforms can be variable, an attention check question was included towards the end of the survey. In the instructions at the top of the questionnaire, participants were told to fill in the word “green” as their favorite color when the question was posed below. As specified in our pre-registration, anyone who answered a color other than green (*n* = 162) was excluded from analyses.

### Data analytic plan

All statistical analyses were conducted using RStudio (version 1.4.1717). Continuous variables of interest (descriptive norms, injunctive norms, contact quantity, contact quality, perceived weight, ingroup identification, explicit weight bias, political orientation, and BMI) were examined for outliers and for normality. We identified outliers using the Median Absolute Deviation (MAD) method, which has been shown to be a favorable, robust alternative to other outlier-identifying methods (e.g., boxplots, plus or minus three standard deviations; [68]). We found and removed outlier values in the injunctive norms variable (*n* = 6), contact quality variable (*n* = 2), explicit weight bias variable (*n* = 12), and BMI variable (*n* = 11). For the BMI variable, we excluded all outliers identified by the MAD method and any BMI values < 10, which are considered biologically implausible [69]. After removal of outliers, all values of skewness fell between -1 and 1 and histograms appeared approximately normal.

Based on prior work in this area, we tested age, sex, race, region of the United States, political orientation, and BMI as potential covariates for models predicting weight bias [39, 41, 70–72]. Region of the United States was not significantly related to weight bias (*F*(4,254) = 1.44, *p* = 0.22), so it was not included as a covariate. The variables that significantly predicted weight bias (i.e., age, sex, political orientation, BMI) were included as covariates in models predicting weight bias (see Table 2 for descriptive statistics). One exception was that BMI was not included as a covariate in models that also included perceived weight as a predictor of weight bias, which was a decision specified in our pre-registration. Because we did not find a strong justification to do so in the literature, we did not include any covariates in models predicting descriptive norms or ingroup identification. Each of these decisions was made a priori, and for all analyses, we used the alpha criterion *p* < 0.05.

**Table 2:** Descriptive statistics and bivariate correlations of study variables.

	1.	2.	3.	4.	5.	6.	7.	8.	9.
1. Descriptive Norms	43.30 (17.48)								
2. Injunctive Norms	-0.14*	5.36 (0.87)							
3. Contact Quantity	0.45***	-0.09	3.63 (1.36)						
4. Contact Quality	< 0.01	-0.02	0.32***	5.56 (1.03)					
5. Perceived Weight	0.24***	-0.1	0.29***	0.14*	4.11 (1.35)				
6. Ingroup Identification	-0.04	0.11	0.01	-0.08	-0.06	4.17 (1.3)			
7. BMI	0.28***	-0.06	0.28***	0.10	0.79***	-0.03	25.33 (5.32)		
8. Political Orientation	0.07	-0.07	-0.02	-0.29***	-0.11	0.02	-0.04	2.67 (1.63)	
9. Explicit Weight Bias	-0.18**	0.13*	-0.37***	-0.56***	-0.13*	0.05	-0.15*	0.22***	1.54 (1.26)

*Note*. Mean (standard deviation) are reported along the diagonal, and correlation coefficients are reported below the diagonal. Significance indicated by

*p ≤ 0.05

**p ≤ 0.01

***p ≤ 0.001.

#### Model summaries

To investigate Hypotheses 1–3, we used three multiple linear regression models to examine the relationships between each proposed predictor (i.e., descriptive norms, contact quantity, contact quality) and explicit weight bias, accounting for pre-registered covariates (i.e., Models 1–3). To test Hypothesis 4, we used simple linear regression to test whether participants’ quantity of contact predicted their descriptive norms about obesity (i.e., Model 4). To explore Hypotheses 5 and 6, we used two multiple linear regression models to test whether injunctive norms or perceived weight, respectively, predicted weight bias controlling for pre-registered covariates (i.e., Models 5 and 6). We used three multiple linear regression models to examine Hypothesis 7 (i.e., Models 7a, 7b, 7c). For each perceived weight subgroup (i.e., heavy, average, thin), we tested whether ingroup identification predicted weight bias. Finally, we tested Hypothesis 8 using simple linear regression to see whether injunctive norms would positively predict ingroup identification among individuals who perceived themselves as heavy (i.e., Model 8).

#### Pre-registration and data

Study methods, hypotheses, and analyses were pre-registered via the Open Science Framework prior to beginning data collection. We also made some small updates to the study plan during data collection, which we summarized and registered via the Open Science Framework as well. In addition, after conducting our confirmatory analyses, we decided to add two *post hoc* tests of the influence of contact quality, contact quantity, and descriptive norms on explicit weight bias. The purpose of these additional tests was to explore the relative strength of these predictors and to gain insight into what might be useful to focus on in future efforts to reduce weight bias. Study materials and data are available on Open Science Framework, and R scripts are available upon request.

## Results

### Pre-registered analyses

#### Descriptive norms, contact, and weight bias (Hypotheses 1–4)

Participants’ perceived descriptive norms significantly negatively predicted their level of explicit weight bias (*b* = -0.01, *p* < 0.01, 95% CI [-0.022, -0.004]), indicating that the more common people believed obesity to be, the less weight bias they exhibited (supporting Hypothesis 1). In addition, consistent with Hypotheses 2 and 3, respectively, we found that both quantity of contact (*b* = -0.25, *p* < 0.001, 95% CI [-0.36, -0.14]) and quality of contact (*b* = -0.63, *p* < 0.001, 95% CI [-0.78, -0.49]) were significant, negative predictors of explicit weight bias. Also as predicted (Hypothesis 4), participants’ quantity of contact was significantly positively related to their reported descriptive norms regarding obesity (*b* = 5.86, *p* < 0.001, 95% CI [4.48, 7.23.]). See Table 3 for full model statistics.

**Table 3:** Model statistics for confirmatory multiple linear regression models (Models 1–8).

		**Outcome: Explicit Weight Bias**				
	**Predictor**	*df*	b	SE b	β	Adjusted *R*^2^
*Model 1*	Descriptive Norms	237	-0.01**	< 0.01	-0.18	0.12
*Model 2*	Contact Quantity	237	-0.25***	0.06	-0.26	0.17
*Model 3*	Contact Quality	237	-0.63***	0.07	-0.49	0.32
*Model 5*	Injunctive Norms	234	-0.25*	0.1	-0.16	0.1
*Model 6*	Perceived Weight	254	-0.14*	0.06	-0.15	0.08
	Ingroup Identification					
*Model 7a*	Among “Heavy” Participants	85	-0.04	0.09	-0.05	0.09
*Model 7b*	Among “Average” Participants	95	-0.03	0.11	-0.02	0.12
*Model 7c*	Among “Thin” Participants	64	0.19	0.1	0.22	0.06
		**Outcome: Descriptive Norms**				
*Model 4*	Contact Quantity	270	5.86***	0.7	0.45	0.2
		**Outcome: Ingroup Identification**				
*Model 8*	Injunctive Norms					
	Among “Heavy” Participants	91	-0.14	0.15	-0.1	< 0.01

*Note*. Models 1, 2, 3, and 5 include age, sex, political orientation, and BMI as covariates. Model 6 includes age, sex, and political orientation as covariates. Models 7a-7c are stratified by participants’ perceived weight, and Model 8 only includes participants with a “heavy” perceived weight. All of these decisions were pre-registered. Significance indicated by

*p ≤ 0.05

**p ≤ 0.01

***p ≤ 0.001.

#### Injunctive norms, ingroup favoritism, and weight bias (Hypotheses 5–8)

Participants’ reported injunctive norms significantly negatively predicted explicit weight bias (*b* = -0.25, *p* = 0.01, 95% CI [-0.44, -0.06]), such that the more people believed that other people approve of obesity, the less weight bias they exhibited (supporting Hypothesis 5). We also found evidence for an ingroup favoritism effect (Hypothesis 6), as participants’ perceived weight significantly negatively predicted their explicit weight bias (*b* = -0.14, *p* = 0.02, 95% CI [-0.26, -0.03]).

To examine whether ingroup identification among heavy individuals predicted lower weight bias, we tested this relationship in the subset of participants who self-reported their weight as “slightly,” “moderately,” or “very” heavy. Among this group (*n*_heavy_ = 95), we failed to find support for Hypothesis 7, namely, that ingroup identification would predict weight bias (*b* = -0.04, *p* = 0.62, 95% CI [-0.22, 0.13]). However, it is notable that mean levels of ingroup identification among this group were about one point below the midpoint of the scale (*M*_heavy_ = 3.99, *SD*_heavy_ = 1.24). We also ran exploratory tests among those who reported “average” or “thin” (i.e., “slightly,” “moderately,” and “very thin”) perceived weights. The mean level of ingroup identification was slightly higher for each of these groups than it was for the “heavy” group: (*n*_average_ = 102, *M*_average_ = 4.28, *SD*_average_ = 1.25; *n*_thin_ = 74, *M*_thin_ = 4.25, *SD*_thin_ = 1.43), but this difference was not significant, (*F*(2, 232) = 1.19, *p* = 0.31). We found no evidence for a relationship between ingroup identification and explicit weight bias among individuals with average perceived weight (*b* = -0.03, *p* = 0.80, 95% CI [-0.25, 0.20]), but this relationship was marginal among individuals with thin perceived weight (*b* = 0.19, *p* = 0.07, 95% CI [-0.02, 0.39]), such that those who identified more strongly with other thin people reported higher weight bias.

Finally, we tested whether perceived injunctive norms would positively predict ingroup identification among individuals who perceived themselves as heavy (Hypothesis 8). We did not find evidence for this prediction (*b* = -0.14, *p* = 0.35, 95% CI [-0.42, 0.15]).

#### *Post hoc* analyses

After reviewing the results of our pre-registered analyses, we decided to probe further into the relative strength of the variables pertaining to contact (i.e., perceived descriptive norms, contact quantity, contact quality) in predicting weight bias. To do so, we conducted a *post hoc* analysis that examined the relevant variables simultaneously. However, because quantity of contact and perceived descriptive norms were significantly correlated (*r* = 0.45, *p* < 0.001) and conceptually similar, we chose not to include both of them in any single regression model. Thus, we conducted one multiple linear regression model using contact quality and contact quantity to predict explicit weight bias (i.e., *post hoc* Model 1) and another using contact quality and perceived descriptive norms to predict explicit weight bias (i.e., *post hoc* Model 2). In each of these *post hoc* models, we included the same covariates used in our pre-registered models predicting weight bias (i.e., age, sex, political orientation, BMI).

We found that contact quality was a considerably stronger predictor of explicit weight bias than contact quantity (*β*s = -0.46 and -0.10, respectively, see Table 4), and that when both variables were included in the regression model (*post hoc* Model 1), only contact quality remained a significant predictor (*b* = -0.58, *p* < 0.001, 95% CI [-0.74, -0.43]). Similarly, we found that contact quality was a stronger predictor of explicit weight bias than was perceived descriptive norms (*β*s = -0.49 and -0.08, respectively), and that only contact quality was a significant predictor (*b* = -0.63, *p* < 0.001, 95% CI [-0.77, -0.49]) when both were included in the regression model (*post hoc* Model 2).

**Table 4:** Model statistics for *post hoc* models predicting explicit weight bias.

	Outcome: Explicit Weight Bias				
	Model Statistics		Coefficients		
Predictors	*df*	Adjusted *R*^2^	b	SE b	β
*Post Hoc* Model 1	236	0.33			
Contact Quality			-0.59***	0.08	-0.46
Contact Quantity			-0.10^+^	0.06	-0.10
Age			0.01^+^	0.01	0.11
Sex			-0.36*	0.16	0.13
BMI			-0.02	0.01	-0.08
Political Orientation			0.04	0.05	0.05
*Post Hoc* Model 2	236	0.32			
Contact Quality			-0.63***	0.07	-0.49
Descriptive Norms			< 0.01	< 0.01	-0.09
Age			0.01*	< 0.01	0.12
Sex			-0.32*	0.16	0.11
BMI			-0.02	0.01	-0.08
Political Orientation			0.04	0.05	0.05

*Note*. Sex was coded such that 0 = Male and 1 = Female. Significance indicated by

^+^*p* ≤ 0.1

*p ≤ 0.05

**p ≤ 0.01

***p ≤ 0.001.

## Discussion

The current findings provide support for our predictions regarding the role of perceived descriptive norms and intergroup contact in weight stigma. We found that participants’ perceived descriptive norms, quantity of contact, and quality of contact with respect to the concept of obesity negatively predicted their level of weight bias. In addition, we found support for the hypothesis that these concepts are closely linked, such that the more contact participants experienced with people with higher weight, the more common they believed obesity to be. These results are consistent with the literature on intergroup contact, as well as the findings from some studies of weight stigma and contact [39, 40].

Our findings also provide a potential explanation as to why the prevalence of weight stigma has not notably decreased in recent decades despite increases in population BMI (and, presumably, increased contact with people with higher weight). The results from our *post hoc* analyses suggest that *quality* of contact is a more important predictor of lower weight bias than either quantity of contact or perceived descriptive norms, that is, how common individuals believe it is to have a higher weight. Therefore, any increased frequency of brief, superficial interactions with higher-weight individuals that has resulted from the increased prevalence of obesity may not be sufficient to influence weight bias or anti-fat attitudes. To reduce weight bias, it may be crucial to focus on experiences of high-quality contact with individuals with higher weight, but further experimental work is needed to clarify the nature of this finding.

It is important to note that our measure of contact quality was not focused on closeness of people’s relationships with people with higher weight; rather, it centered on the nature of the contact itself. Specifically, our measure of contact quality [32] assessed the extent to which people perceived their contact with people with higher weight as “equal,” “voluntary,” “pleasant,” “cooperative,” and not “superficial.” These aspects of contact were drawn from Allport’s [33] predictions concerning the conditions under which intergroup contact may lead to more positive intergroup attitudes. Thus, it is not necessarily surprising that contact quality was the most important predictor of lower weight bias in this sample. Nonetheless, these findings suggest potential interventions designs to reduce weight bias. For instance, future studies might examine ways that people could pay more attention to positive aspects of their interactions with individuals with higher weight (e.g., through guided reflection, self-affirmation, or imagined contact), and they might help create opportunities for people to experience interactions with people with higher weight that include the key features of a high-quality interaction (e.g., equal status, voluntary, cooperative). These types of interventions might be particularly useful for people who frequently interact with higher-weight people but who tend to have high levels of weight bias, such as healthcare providers [73]. Our findings also underscore that when conducting research on weight stigma, it is important to measure both quantity and quality of contact, as each can uniquely contribute to our understanding of the usefulness of contact for reducing weight bias.

We also found support for most of our hypotheses pertaining to injunctive norms and ingroup favoritism. As predicted, reported injunctive norms regarding obesity and perceived weight were negatively related to weight bias. These findings suggest that people’s perceptions of others’ approval (or disapproval) of having a high weight influences their own weight bias, and that ingroup favoritism exists in the context of weight bias. This latter finding may help to clarify some of the mixed findings in the area of ingroup favoritism and weight stigma, given that prior relevant research relied on BMI rather than perceived weight to predict weight bias [39, 47, 50, 51]. Because not all individuals who have high BMIs perceive themselves as heavy [74], it is not surprising that tests of the relationship between BMI (rather than perceived weight) and explicit weight bias have produced inconclusive results.

One prediction that was not confirmed in this study was the expected positive relationship between reported injunctive norms and ingroup identification among individuals who perceived themselves as heavy. Our rationale for this prediction was that individuals with high perceived weight might be more willing to identify with a heavy ingroup if they believed that other people were more approving of having a higher weight, but this was not the case.

We also found little support for our hypotheses regarding the role of ingroup identification in explicit weight bias. We expected that people who perceive themselves as heavy and who strongly identify with higher-weight people as their ingroup would show particularly low levels of weight bias. However, this hypothesis was not supported; nor did we find evidence for this relationship in our exploratory tests among self-perceived “thin” or “average weight” participants. This null result may have reflected the fact that participants did not report much ingroup identification with their perceived weight groups in the first place (i.e., all group means were below the “neutral” midpoint). Thus, there may not have been enough meaningful variability in ingroup identification to test our hypotheses. Future research should employ different measures of ingroup identification and/or explore whether people identify more strongly with other words used to describe their body size. Aside from these issues, one possible explanation for why ingroup favoritism was unrelated to ingroup identification can be drawn from Hinkle and Brown [75], who assert that a negative relationship between ingroup identification and bias can only be expected if group members feel both connected to their ingroup and concerned about their group’s status relative to other groups. It is possible that self-perceived “heavy” individuals with strong ingroup identification do not necessarily feel concerned about how their ingroup fares relative to “thin” or “average weight” groups; however, we did not collect data about these concerns in the present study. We also did not measure internalized weight bias in this study, but doing so in the future might provide insight into the relationship between ingroup identification and weight bias. Indeed, one study found that internalized weight bias was an important moderator of the relationship between social identification and stigma-related distress [76], suggesting that people with high internalized weight bias may not benefit as much from social identification as those with low internalized bias. Perhaps ingroup identification is related to ingroup favoritism, but only among those with low internalized weight bias. Alternatively, it is possible that weight bias is so strong in present-day society that no amount of ingroup identification is sufficient to overcome it. Regardless, it will be important for future research to examine this relationship further.

### Limitations and future directions

Limitations associated with this study are worth noting. First, the cross-sectional survey design and correlational nature of our analyses prevented us from drawing causal conclusions or asserting directional claims. We chose to focus on the degree to which people’s pre-existing perceptions of social norms, contact with people with higher weight, and perceived weight influence their degree of weight bias, and these views could obviously not be randomly assigned. Studies that manipulate information about social norms or that randomly assign participants to varying amounts of high-quality contact with people with higher weight (e.g., [42]) will be useful to better understand these relationships. In addition, we used a sample from Prolific, an online survey platform, and Prolific participants are not necessarily representative of the general population [77]. Indeed, our sample was largely white as well as somewhat thinner than the U.S. population as a whole, which limits our ability to generalize our findings. It is important for future work on this topic to explore these relationships along with racial/ethnic identity and socioeconomic status, as both are related to expressions and experiences of weight bias [78, 79]. Finally, we note that data collection took place in July of 2021, during the SARS-CoV-2 (COVID-19) pandemic. Although most COVID-related lockdowns and travel restrictions had lifted by that time in the United States, this context may have exerted an influence on people’s social contact with higher-weight people and weight-related attitudes. Thus, it will be important for future research to replicate and extend these findings in other contexts.

## Conclusions

To our knowledge, this study is the first to integrate the concepts of descriptive and injunctive norms regarding obesity with the concepts of intergroup contact and ingroup favoritism in the context of weight stigma. Our cross-sectional survey findings suggest that both the perceived prevalence and social acceptability of having a higher weight are associated with people’s explicit weight bias, and that intergroup contact and ingroup favoritism generally operate as expected in the context of weight. Social psychologists are uniquely positioned to examine the issue of weight stigma, and future work will be needed to understand the beliefs and motivations that underlie weight bias and weight-related social norms. Ultimately, we intend for this research to generate further examination of the pervasiveness of negative attitudes toward people with higher weight, particularly as it relates to strategies to reduce widespread weight stigma.
